# Biotransformation of steroids by entomopathogenic strains of *Isaria farinosa*

**DOI:** 10.1186/s12934-018-0920-0

**Published:** 2018-05-12

**Authors:** Ewa Kozłowska, Natalia Hoc, Jordan Sycz, Monika Urbaniak, Monika Dymarska, Jakub Grzeszczuk, Edyta Kostrzewa-Susłow, Łukasz Stępień, Elżbieta Pląskowska, Tomasz Janeczko

**Affiliations:** 1 Department of Chemistry, Wrocław University of Environmental and Life Sciences, Norwida 25, 50-375 Wrocław, Poland; 2 Department of Pathogen Genetics and Plant Resistance, Institute of Plant Genetics, Polish Academy of Sciences, Strzeszyńska 34, 60-479 Poznań, Poland; 3 Department of Plant Protection, Plant Pathology and Mycology Division, Wrocław University of Environmental and Life Sciences, pl. Grunwaldzki 24a, 50-363 Wrocław, Poland

**Keywords:** *Isaria farinosa*, Biotransformation, DHEA, Progesterone, Hydroxylation

## Abstract

**Background:**

Steroid compounds are very interesting substrates for biotransformation due to their high biological activity and a high number of inactivated carbons which make chemical modification difficult. Microbial transformation can involve reactions which are complicated and uneconomical in chemical synthesis, and searching for a new effective biocatalyst is necessary. The best known entomopathogenic species used in steroid modification is *Beauveria bassiana*. In this study we tested the ability of *Isaria farinosa*, another entomopathogenic species, to transform several steroids.

**Results:**

Twelve strains of the entomopathogenic filamentous fungus *Isaria farinosa*, collected in abandoned mines located in the area of the Lower Silesian Voivodeship, Poland, from insects’ bodies covered by fungus, were used as a biocatalyst. All the tested strains effectively transformed dehydroepiandrosterone (DHEA). We observed 7α- and 7β-hydroxy derivatives as well as changes in the percentage composition of the emerging products. Due to the similar metabolism of DHEA in all tested strains, one of them was selected for further investigation. In the culture of the selected strain, *Isaria farinosa* KCh KW1.1, transformations of androstenediol, androstenedione, adrenosterone, 17α-methyltestosterone, 17β-hydroxyandrost-1,4,6-triene-3-one and progesterone were performed. All the substrates were hydroxylated with high yield and stereoselectivity. We obtained 6β-hydroxyandrost-4-ene-3,11,17-trione, 15α,17β-dihydroxy-6β,7β-epoxyandrost-1,4-diene-3-one and 6β,11α-dihydroxyprogesterone. There is no evidence of either earlier microbial transformation of 17β-hydroxyandrost-1,4,6-triene-3-one or new epoxy derivatives.

**Conclusions:**

*Isaria farinosa* has a broad spectrum of highly effective steroid hydroxylases. The obtained 7-hydroxydehydroepiandrosterone has proven high biological activity and can be used in Alzheimer’s disease and as a key intermediate in the synthesis of aldosterone antagonists. Transformation of progesterone leads to high yield of 6β,11α-dihydroxyprogesterone and it is worth further study.

## Background

Biotransformation is an alternation of a xenobiotic substrate within an organism, as a result of its enzymatic activity. Enzymes can catalyse a wide spectrum of reactions like hydroxylation in inactivated positions. Microorganisms can perform reactions which are complicated and economically unprofitable as chemical synthesis because of their high regio-, chemo-, stereo and enantioselectivity [1–4]. Some specific microbial transformations have been successfully incorporated into numerous syntheses of steroid drugs and key intermediates [2, 3, 5–7]. Applications of microorganism for steroid biotransformation have attracted considerable attention in the steroid chemistry community. In the literature, there are well-documented steroid transformations such as hydroxylation, dehydrogenation, redox reaction or Baeyer–Villiger oxidation with numerous microorganisms [7–11]. Among them, hydroxylation of steroid compounds by filamentous fungi is a key reaction to obtain hydroxylated derivatives with higher biological properties than their precursors [5, 12, 13]. The use of biotransformation methods is superior to chemical synthesis due to the difficulty of attaching an oxygen atom to an inactivated carbon atom in the steroid structure.

Through the development of biotransformation, it has been possible to develop methods for obtaining high yields of functionalized steroid compounds widely used in commercial production such as corticosteroids [14]. However, successful application of biotransformation for the industrial synthesis of known and novel steroids is still limited [7]. Therefore, further screening for new active catalytic strains is recommended.

The species *Isaria farinosa* belongs to the Cordycipitaceae family in the Hypocreales order of Ascomycota. Many of the representatives of this family are parasitic, mainly infecting insects [15–17]. In recent years some of them, like *Beauveria, Metarhizium* and less frequently *Isaria* are used in biotransformation. *Beauveria bassiana* is well-known biocatalyst for the transformation of steroid compounds. Hydroxylations at the C-7α and C-11α position and Baeyer–Villiger oxidation to d-homo lactones are described in the literature [18–24]. Also *Metarhizium anisopliae* have ability of hydroxylation at 6β and 11α position [25]. The catalytic activity of *Isaria fumosorosea* towards selected steroid compounds with high substrate specificity was described [26]. The high virulence of *Isaria farinosa*, as other mentioned entomopathogenic strains, is closely connected with high chitinase, lipase and protease activities [12]. For this reasons, *Isaria farinosa* strains were used as biocatalysts in the biotransformation of steroid compounds for the first time. The high activity and specificity of the dehydrogenases and hydroxylases present in their cells were also demonstrated.

## Methods

### Materials

**Fig. 1:**
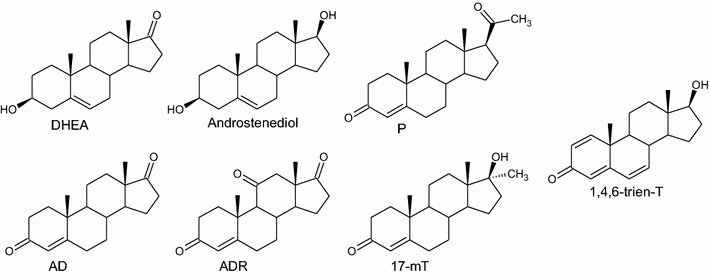
Structures of substates

The following substrates were purchased from Sigma-Aldrich: dehydroepiandrosterone (DHEA), androstenediol (androst-5-ene-3β,17β-diol), androstenedione (androst-4-ene-3,17-dione, AD), adrenosterone (androst-4-ene-3,11,17-trione, ADR), progesterone (P), 17α-methyltestosterone (17-mT) (Fig. 1.). 17β-Hydroxyandrost-1,4,6-triene-3-one (1,4,6-triene-T) was taken from the resources of the Department of Chemistry, Wrocław University of Environmental and Life Sciences.

Microorganisms: Twelve *Isaria farinosa* strains named KCh J1.1, KCh J1.2, KCh J1.3, KCh J1.4, KCh J1.6, KCh J2.2, KCh J2.3, KCh J2.4, KCh J2.6, KCh KW1.1, KCh KW1.2 and KCh RJ1.1 were obtained from the collection of the Department of Chemistry, Wrocław University of Environmental and Life Sciences, Wrocław, Poland. The strains were maintained on Sabouraud 4% dextrose-agar slopes and freshly subcultured before being used in the transformation experiments.

### Isolation of strains

**Fig. 2:**
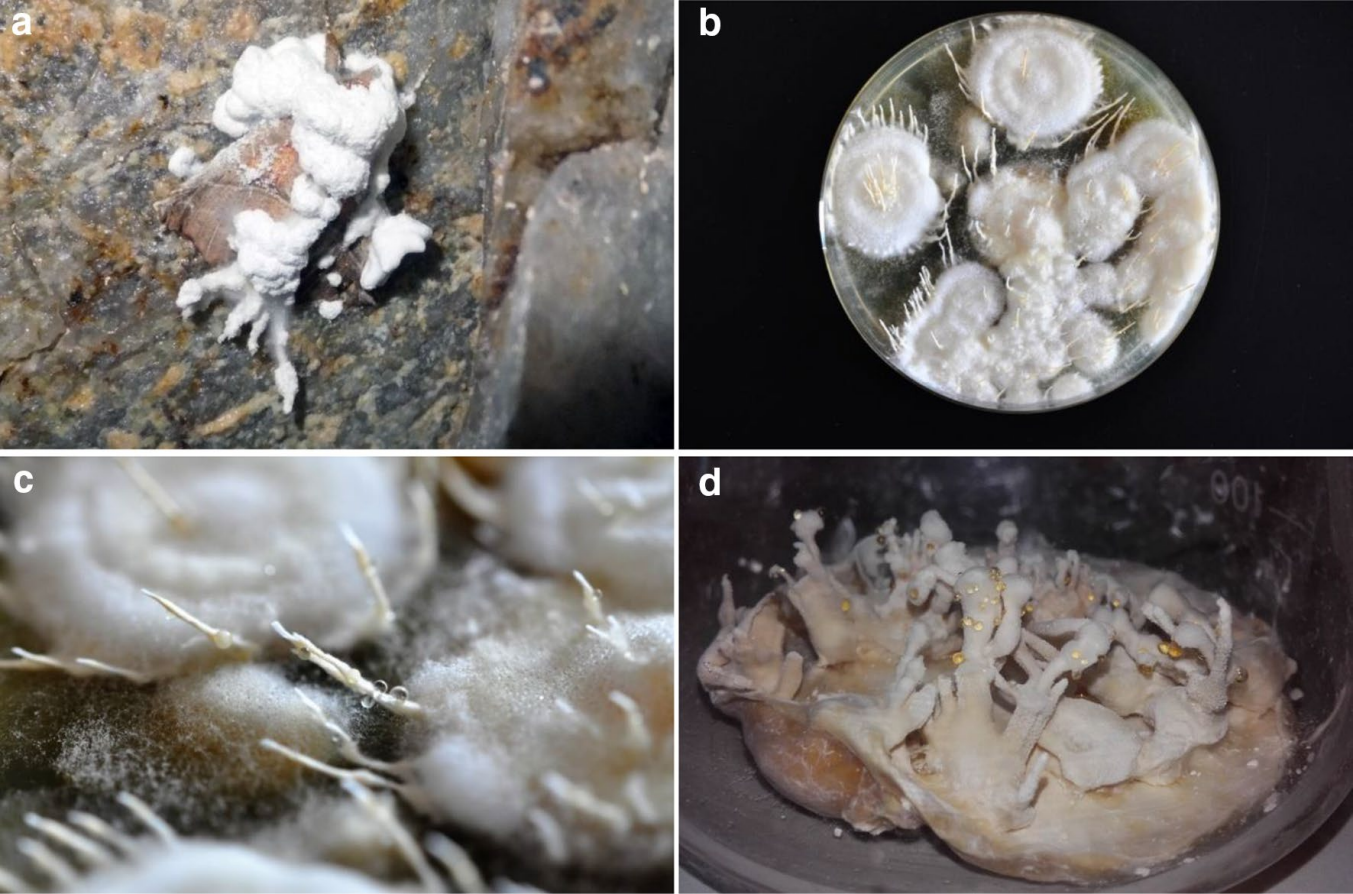
*Isaria farinosa* strains: **a** A moth cadaver found on a wall in an adit near Ciechanowice (Lower Silesian Voivodeship, Poland) overgrown by hyphae of *Isaria farinosa* © Jakub Grzeszczuk; **b** *I. farinosa* on PDA medium © Jakub Grzeszczuk; **c** Synnemata of *I. farinosa* grown on PDA medium © Jakub Grzeszczuk; **d** *I. farinosa* grown on cultivation medium © Tomasz Janeczko

The studied samples were collected in different abandoned mines located in the area of the Lower Silesian Voivodeship, Poland. The samples named as KCh J*.* were collected in an adit near Ciechanowice, the samples marked as KCh KW*.* were collected in an adit near Kletno, and those named as KCh RJ*.* were collected in an adit in the area of the Izera Crossroads in the Izera Mountains. The fungi were collected from these sites in the months of winter 2014/2015. After entering the adits, bodies of insects covered by fungal growths were searched for on the walls of the mines’ corridors. When found, cadavers of insects overgrown by the fungal hyphae were placed in sterile plastic containers with the help of previously sterilized tweezers. Afterwards, the samples were transported to the laboratory of the Plant Pathology and Mycology Division of the Department of Plant Protection, Wrocław University of Environmental and Life Sciences. There, pieces of hyphae from each sample were carefully pulled out from the insect’s cadavers and placed on Petri dishes with PDA medium. The growth of the fungi was observed for 4 weeks (Fig. 2).

### DNA extraction and molecular identification of fungal strains

Growing mycelia of individual fungal strains were maintained in pure cultures for 7 days on PDA medium for genomic DNA extraction. Genomic DNA was extracted using a modified CTAB (hexadecyltrimethylammonium bromide) method, described earlier [27]. The DNA extracts were stored at – 20 °C until used. Species identification was done on the basis of the sequences of the Internal Transcribed Spacers of the ribosomal DNA region (ITS1-ITS2).

Polymerase chain reactions were performed as described previously by Kozłowska et al. [9], using DreamTaq Green DNA polymerase (Thermo Scientific, Espoo, Finland). The PCR amplification was done using ITS4 (5′-TCCTCCGCTTATTGATATGC-3′) and ITS5 (5′-GGAAGTAAAAGTCGTAACAAGG-3′) primers. Amplicons were electrophoresed in 1.5% agarose gel (Invitrogen) with GelGreen Nucleic Acid Stain (Biotium, Inc.).

For sequence analysis, PCR-amplified DNA fragments were purified as described previosly by Kozłowska et al. [9], DNA fragments were labelled using a forward primer and the BigDyeTerminator 3.1 kit (Applied Biosystems, Foster City, CA, USA), according to the producer’s recommendations, and precipitated with 96% ethanol. Sequence reading was performed using Applied Biosystems equipment. Sequences were analysed using the BLASTn algorithm.

### Screening procedure

Erlenmeyer flasks (300 ml), each containing 100 ml of the cultivation medium (3% glucose, 1% bacteriological peptone), were inoculated with a suspension of the investigated strain and then incubated for 3 days at 24 °C on a rotary shaker. Then 10 mg of the substrate dissolved in 1 ml of THF was added. Samples were taken at 3, 6, 9, 12 h (only for DHEA as a substrate) and on the 1st, 3rd and 7th day of the process (for all of the substrates) and products were subsequently extracted using chloroform and analysed using TLC and GC.

### Preparative biotransformation

The same transformations were performed on the preparative scale in 2000 ml flasks, each containing 500 ml of the cultivation medium. The culture of *I. farinosa* KCh KW1.1 was incubated under the same conditions as in the screening procedure and then 100 mg of substrate dissolved in 2 ml of THF was added to the 3-day-old culture. After the complete transformation of the substrate, the mixture was extracted with CHCl_3_ (3 × 300 ml), dried (MgSO_4_) and concentrated *in vacuo*. The crude mixture obtained this way was separated by preparative TLC and analysed (TLC, GC).

### Analytical methods

The course of the biotransformation was controlled by means of TLC. The composition of product mixtures was established by GC. The crude mixture was separated by preparative TLC (Silica Gel GF, 500 μm, Analtech) with a hexane/acetone mixture (2:1, v/v) as the eluent. After elution products were detected under UV light (365 nm), then scraped from the plate and eluted with acetone to give fractions. Analytical TLC was carried out on silica gel G (Merck). Compounds were detected by spraying the plates with a H_2_SO_4_/CH_3_OH mixture (1:1, v/v) and visualized under UV light (254 nm). GC analysis was performed using a Hewlett-Packard 5890A (Series II) GC instrument fitted with a flame ionization detector (FID). The DB-5MS (cross-linked phenyl methyl siloxane) capillary column (30 m × 0.32 mm × 0.25 μm) was used to determine the composition of product mixtures. The following temperature program was used: 250 °C (1 min)/5 °C/min/300 °C (6 min). The NMR spectra were recorded on a DRX 500 MHz Bruker spectrometer and measured in CDCl_3_. The products’ structures were determined by means of elemental analysis, ^1^H NMR, ^13^C NMR and correlation spectroscopy (HMBC, HSQC).

## Results and discussion

### Strain identification

The colonies of *I. farinosa* grew relatively slowly on the PDA medium, appearing smooth and delicate, soon forming white or yellowish synnemata which were up to 50 mm long (Fig. 2). The fungi were also observed under a microscope. Aerial hyphae were hyaline, quite loose, with smooth, thin walls. Conidiogenous cells were scattered, growing out directly from general hyphal mass or from side branches, sporadically present in dense clusters. Conidia were single-celled, hyaline, smooth, with thin walls. No chlamydospores were noted.

The twelve fungal strains used in this study were identified by determination of the ITS1-ITS2 sequence. The DNA fragments were amplified using PCR with ITS4 -ITS5 primers and sequenced. The complete sequences of these products were compared with reference ITS sequences deposited in the GenBank database (Table 1).

**Table 1:** Identification of fungal strains on the basis of the sequence of the ITS1-ITS2 sequences, and comparison with reference ITS sequences

Name of fungal strain	Identified fungal species	Sequence identity
KCh J1.1	*Isaria farinosa*	99% identity to *Isaria farinosa*, Acc. Numbers: HQ115724.1, AB080087.1, GU354351.1
KCh J1.2	*Isaria farinosa*	99% identity to *Isaria farinosa*, Acc. Numbers: MF950892.1, KX882684.1, MF326609.1
KCh J1.3	*Isaria farinosa*	99% identity to *Isaria farinosa*, Acc. Numbers: HQ115724.1, AB080087.1, GU354351.1
KCh J1.4	*Isaria farinosa*	98% identity to *Isaria farinosa*, Acc. Numbers: MF950892.1, MF326609.1, KT368167.1
KCh J1.6	*Isaria farinosa*	99% identity to *Isaria farinosa*, Acc. Numbers: MF950892.1, MF326609.1, KT368167.1
KCh J2.2	*Isaria farinosa*	98% identity to *Isaria farinosa*, Acc. Numbers: HQ115724.1, KU214569.1, KU214564.1
KCh J2.3	*Isaria farinosa*	99% identity to *Isaria farinosa*, Acc. Numbers: HQ115724.1, AB080087.1, KU214569.1
KCh J2.4	*Isaria farinosa*	99% identity to *Isaria farinosa*, Acc. Numbers: HQ115724.1, GU354351.1, AB080087.1
KCh J2.6	*Isaria farinosa*	99% identity to *Isaria farinosa*, Acc. Numbers: HQ115724.1, GU354351.1, AB080087.1
KCh KW1.1	*Isaria farinosa*	100% identity to *Isaria farinosa*, Acc. Numbers: MF950892.1, KX882684.1, MF326609.1
KCh KW1.2	*Isaria farinosa*	86% identity to *Isaria farinosa*, Acc. Numbers: HE664156.1, HQ907945.1, MF950892.1
KCh RJ1.1	*Isaria farinosa*	99% identity to *Isaria farinosa*, Acc. Numbers: MF950892.1, MF326609.1, KT368167.1

The phylogenetic analysis did not show any significant differences between investigated fungal strains. Also in comparison with fungal strains of *Isaria farinosa* isolated from different environments and deposited in the collection of the Institute of Plant Genetics, Polish Academy of Sciences, Poznań, Poland, no differences in sequences were observed.

### Spectral data of isolation metabolites

#### Transformation of adrenosterone (ADR)

After 3-day incubation of 100 mg of adrenosterone in *Isaria farinosa* KCh KW1.1 culture 30.1 mg (30%) of 6β-hydroxyandrost-4-ene-3,11,17-trione (6β-OH-ADR) was isolated as the main product.

##### Spectral data of isolated 6β-hydroxyandrost-4-ene-3,11,17-trione (6β-OH-ADR)

^1^H NMR (600 MHz) (ppm) (CDCl3) *δ*: 0.89 (s, 3H, 18-H); 1.50 (ddd, 1H, *J* = 14.1, 11.6, 2.6 Hz, 7-Hα); 1.60 (s, 3H, 19-H); 1.62 (td, 1H, *J* = 14.5, 4.0 Hz, 1-Hα); 1.73 (ddd, 1H, *J* = 21.4, 12.1, 9.4 Hz, 15-Hβ); 1.86 (d, 1H, *J* = 11.5 Hz, 9-H); 1.89 (td, 1H, *J* = 11.8, 5.7 Hz, 14-H); 2.12–2.18 (m, 1H, 15-Hα); 2.20–2.37 (m, 4H, 2-Hα, 7-Hβ, 12-Hα, 16-Hα); 2.44–2.60 (m, 4H, 2-Hβ, 8-H, 12-Hβ, 16-Hβ); 2.80 (dm, 1H, *J* = 13.4 Hz, 1-Hβ); 4.39 (br s, 1H, 6-Hα); 5.79 (br s, 1H, 4-H).

#### Transformation of progesterone (P)

After 3-day incubation of 100 mg of progesterone in *Isaria farinosa* KCh KW1.1 culture 32.9 mg (33%) of 6β,11α-dihydroxyprogesterone (6β,11α-OH-P) and 4.1 mg (4%) of 6β-hydroxy-11-oxo-progesterone (6β-OH,11-oxo-P) were isolated.

##### 6β,11α-dihydroxyprogesterone (6β,11α-OH-P)

^1^H NMR (600 MHz) (ppm) (CDCl3) *δ*: 0.73 (s, 3H, 18-H); 1.10 (t, 1H, *J* = 10.3 Hz, 9-H); 1.27–1.32 (m, 2H, 14-H, 15-Hβ); 1.50 (s, 3H, 19-H); 1.51 (t, 1H, *J* = 11.4 Hz, 12-Hα); 1.67–1.78 (m, 2H, 15-Hα, 16-Hα); 1.92 (td, 1H, *J* = 14.4, 4.4 Hz, 1-Hα); 1.99 (dt, 1H, *J* = 13.9, 3.0 Hz, 7-Hα); 2.01–2.07 (qd, 1H, *J* = 11.9, 2.7 Hz, 8-H); 2.13 (s, 3H, 21-H); 2.16–2.23 (m, 1H, 16-Hβ); 2.28–2.40 (m, 3H, 2-Hα, 7-Hβ, 12-Hβ); 2.51–2.58 (m, 2H, 2-Hβ, 17-Hα); 2.79 (ddd, 1H, *J* = 13.7, 4.8, 3.0 Hz, 1-Hβ); 4.09 (td, 1H, *J* = 10.6, 4.8 Hz, 11-Hβ); 4.34 (t, 1H, *J* = 2.8 Hz, 6-Hα); 5.81 (s, 1H, 4-H).

##### 6β-hydroxy-11-oxo-progesterone (6β-OH,11-oxo-P)

^1^H NMR (600 MHz) (ppm) (CDCl3) *δ*: 0.67 (s, 3H, 18-H); 1.42–1.52 (m, 2H, 7-Hα, 15-Hβ); 1.60 (s, 3H, 19-H); 1.63 (td, 1H, *J* = 14.3, 3.9 Hz, 1-Hα); 1.73–1.82 (m, 2H, 14-H, 15-Hα); 1.84–1.93 (m, 2H, 9-H, 16-Hα); 2.11 (s, 3H, 21-H); 2.11–2.13 (m, 1H, 7-Hβ); 2.23–2.27 (m, 1H, 16-Hβ); 2.30–2.44 (m, 2H, 2-Hα, 8-H); 2.48 (d, 1H, *J* = 12.3 Hz, 12-Hα); 2.57 (ddd, 1H, *J* = 19.8, 16.1, 6.3 Hz, 2-Hβ); 2.69 (d, 1H, *J* = 12.5 Hz, 12-Hβ); 2.73 (t, 1H, *J* = 10.8 Hz, 17-Hα); 2.83 (ddd, 1H, *J* = 13.6, 5.0, 2.9 Hz, 1-Hβ); 4.37 (t, 1H, *J* = 2.7 Hz, 6-Hα); 5.82 (s, 1H, 4-H).

#### Transformation of 17β-hydroxyandrost-1,4,6-triene-3-one (1,4,6-triene-T)

Three day transformation of 100 mg of 17β-hydroxyandrost-1,4,6-triene-3-one in *Isaria farinosa* KCh KW1.1 culture gave: 18.6 mg (19%) of 15α,17β-dihydroxy-6β,7β-epoxyandrost-1,4-diene-3-one (15α-OH-6,7β-epoxy-1-ene-T), 5.4 mg (5%) of 15α,17β-dihydroxy-6β,7β-epoxyandrost-4-ene-3-one (15α-OH-6,7β-epoxy-T) and 8.2 mg (8%) of 14α,17β-dihydroxy-6β,7β-epoxyandrost-1,4-diene-3-one (14α-OH-6,7β-epoxy-1-ene-T)

##### 17β-hydroxy-androst-1,4,6-triene-3-one (1,4,6-triene-T)

^1^H NMR (600 MHz) (ppm) (CDCl3) *δ*: 0.87 (s, 3H, 18-H); 1.14 (td, 1H, *J* = 12.8, 4.5 Hz, 12-Hα); 1.17–1.23 (m, 1H, 14-H); 1.20 (s, 3H, 19-H); 1.44 (ddd, 1H, *J* = 13.1, 9.8, 3.5 Hz, 9-H); 1.46–1.56 (m, 2H, 15-Hβ, 16-Hβ); 1.65 (qd, 1H, *J* = 12.9, 4.0 Hz, 11-Hβ); 1.78 (ddd, 1H, *J* = 10.5, 7.3, 2.6 Hz, 15-Hα); 1.83 (dq, 1H, *J* = 13.2, 3.7 Hz, 11-Hα); 1.92 (ddd, 1H, *J* = 12.8, 3.6, 3.1 Hz, 12-Hβ); 2.08–2.16 (m, 1H, 16-Hα); 2.32 (t, 1H, *J* = 10.7 Hz, 8-H); 3.68 (t, 1H, *J* = 8.5 Hz, 17-Hα); 5.99–6.02 (m, 2H, 4-H, 7-H); 6.23 (dd, 1H, *J* = 10.1, 2.6 Hz, 6-H); 6.25 (dd, 1H, *J* = 10.1, 1.7 Hz, 2-H); 7.07 (d, 1H, *J* = 10.1 Hz, 1-H).

##### 15α,17β-dihydroxy-6β,7β-epoxyandrost-1,4-diene-3-one (15α-OH-6,7β-epoxy-1-ene-T)

^1^H NMR (600 MHz) (ppm) (CDCl3) *δ*: 1.08 (td, 1H, *J* = 12.7, 4.2 Hz, 12-Hα); 1.12 (s, 3H, 18-H); 1.20 (dd, 1H, *J* = 12.5, 5.8 Hz, 14-H); 1.36 (s, 3H, 19-H); 1.37 (td, 1H, *J* = 11.9, 3.8 Hz, 9-H); 1.67 (td, 1H, *J* = 13.0, 4.2 Hz, 11-Hβ); 1.70 (ddd, 1H, *J* = 14.6, 8.9, 2.6 Hz, 16-Hα); 1.76 (dq, 1H, *J* = 13.3, 3.1 Hz, 11-Hα); 1.85 (ddd, 1H, *J* = 12.8, 3.9, 3.1 Hz, 12-Hβ); 2.55 (t, 1H, *J* = 12.0 Hz, 8-H); 2.67 (ddd, 1H, *J* = 14.7, 8.5, 7.8 Hz, 16-Hβ); 3.54 (d, 1H, *J* = 3.9 Hz, 7-Hβ); 3.59 (d, 1H, *J* = 3.9 Hz, 6-Hβ); 3.62 (t, 1H, *J* = 8.7 Hz, 17-Hα); 4.45 (ddd, 1H, *J* = 8.0, 5.8, 2.6 Hz, 15-Hβ); 6.23 (dd, 1H, *J* = 10.2, 1.9 Hz, 2-H); 6.48 (d, 1H, *J* = 1.9 Hz, 4-H); 6.97 (d, 1H, *J* = 10.2 Hz, 1-H).

##### 15α,17β-dihydroxy-6β,7β-epoxyandrost-4-ene-3-one (15α-OH-6,7β-epoxy-T)

^1^H NMR (600 MHz) (ppm) (CDCl3) *δ*: 1.05–1.12 (m, 2H, 9-H, 12-Hα); 1.09 (s, 3H, 18-H); 1.21 (dd, 1H, *J* = 12.3, 6.2 Hz, 14-H); 1.26 (s, 3H, 19-H); 1.40 (qd, 1H, *J* = 13.1, 3.2 Hz, 11-Hβ); 1.59 (ddd, 1H, *J* = 13.3, 6.8, 3.7 Hz, 11-Hα); 1.64–1.72 (m, 2H, 1-Hβ, 16-Hα); 1.84 (dt, 1H, *J* = 12.7, 3.3 Hz, 12-Hβ); 1.93 (ddd, 1H, *J* = 13.1, 5.0, 2.6 Hz, 1-Hβ); 2.38–2.45 (m, 2H, 2-Hα, 8-H,); 2.61 (ddd, 1H, *J* = 16.7, 14.2, 4.9 Hz, 2-Hβ); 2.68 (ddd, 1H, *J* = 14.5, 8.3, 7.8 Hz, 16-Hβ); 3.40 (d, 1H, *J* = 3.7 Hz, 7-Hα); 3.50 (d, 1H, *J* = 3.7 Hz, 6-Hα); 3.63 (t, 1H, *J* = 8.7 Hz, 17-Hα); 4.46 (ddd, 1H, *J* = 7.9, 5.8, 2.5 Hz, 15-Hβ); 6.17 (d, 1H, *J* = 0.7 Hz, 4-H).

##### 14α,17β-dihydroxy-6β,7β-epoxyandrost-1,4-diene-3-one (14α-OH-6,7β-epoxy-1-ene-T)

^1^H NMR (600 MHz) (ppm) (CDCl3) *δ*: 1.11 (s, 3H, 18-H); 1.27 (td, 1H, *J* = 12.9, 4.2 Hz, 12-Hα); 1.33 (s, 3H, 19-H); 1.56–1.70 (m, 2H, 11-Hβ, 15-Hβ); 1.70–1.83 (m, 2H, 11-Hα, 15-Hα); 1.96 (dd, 1H, *J* = 12.5, 3.5 Hz, 9-H); 2.39–2.49 (m, 2H, 8-H, 16-Hα); 2.50–2.63 (m, 2H, 12-Hβ, 16-Hβ); 3.60 (d, 1H, *J* = 3.9 Hz, 7-Hβ); 3.63 (d, 1H, *J* = 3.9 Hz, 6-Hβ); 6.25 (dd, 1H, *J* = 10.2, 1.8 Hz, 2-H); 6.48 (d, 1H, *J* = 1.8 Hz, 4-H); 6.96 (d, 1H, *J* = 10.2 Hz, 1-H).

### Interpretation of results

The purpose of this study was to investigate the catalytic ability of twelve newly isolated entomopathogenic filamentous fungal strains of *Isaria farinosa* against selected steroid compounds. The strains were isolated from insects’ cadavers and identified by determination of the ITS1-ITS2 rDNA sequence. Substrates in this study were: dehydroepiandrosterone (DHEA), androstenediol, androstenedione (AD), adrenosterone (ADR), progesterone (P), 17α-methyltestosterone (17-mT) and 17β-hydroxyandrost-1,4,6-triene-3-one (1,4,6-triene-T).

**Fig. 3:**
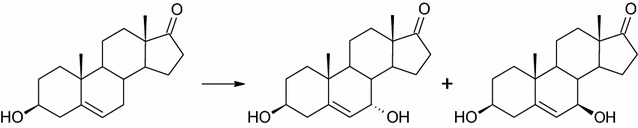
Transformation of DHEA in the culture of *Isaria farinosa* strains. Transformation conditions: 100 mL of cultivation medium (3% glucose, 1% bacteriological peptone) in 300 mL Erlenmeyer flasks, 24 °C, 150 r/min for 7 day

Transformation of dehydroepiandrosterone (DHEA) in the culture of all twelve *Isaria farinosa* strains led to the formation of 7α- and 7β-hydroxy-DHEA (Fig. 3). These products were identified on the basis of identity of their *R*_t_ from GC and *R*_f_ from TLC with standards available in our laboratory [26]. Biotransformation on the preparative scale was redundant. In the screening procedure, seven of them transformed DHEA in less than 24 h, of which three did so in less than 12 h (KCh J2.2, KCh KW1.1 and KCh KW1.2). Prolongation of the process led to degradation of both products. Due to the fast transformation of this substrate modification of the screening procedure was necessary. Analysis of Table 2 indicates that at the beginning phase of transformation the 7α derivative was dominant, but during the process, the reaction mixture changed. According to Lobastova et al., 7α-hydroxylation of DHEA is more often described among filamentous fungi than hydroxylation in the 7β position [28]. In our previous study, a strain of the same species, *Isaria fumosorosea*, hydroxylated DHEA at the 7-position to give two hydroxy derivatives but the 7β formed a majority and subsequent stages of transformation were observed [26]. Production of high yield of 7α-hydroxy-DHEA (7α-OH-DHEA) has great importance due to the high biological activity of this compound and its role as a key intermediate in the synthesis of eplerenone and other aldosterone antagonists [28]. 7α-OH-DHEA has an anti-inflammatory effect and its concentration in all body tissues, including the brain, increases as a body response to inflammatory conditions [29, 30]. The compound reduces the oxidative damage to proteins and lipids and increases the amount of colonic mucus that can be used to treat or prevent colitis [31]. 7-Hydroxy derivatives of DHEA modulate prostaglandin synthesis, inhibit 11β-hydroxysteroid dehydrogenase type 1 (11β-HSD1) and exhibit neuroprotective activity [32–35].

**Table 2:** Composition of the crude mixture obtained in the transformation of DHEA in *Isaria farinosa* strains

*Isaria farinosa* strain	Compounds found in the reaction mixture (%)	Biotransformation time (h)						
		3	6	9	12	24	72	168
KCh J1.1	DHEA	100 ± 0.1	100 ± 0.1	99 ± 0.3	93 ± 0.5	74 ± 3.9	–	–
	7α-OH-DHEA	–	–	–	5 ± 0.7	17 ± 2.7	60 ± 3.0	43 ± 4.6
	7β-OH-DHEA	–	–	–	2 ± 0.5	9 ± 2.3	38 ± 2.6	42 ± 3.8
KCh J1.2	DHEA	100 ± 0.1	100 ± 0.3	72 ± 3.3	39 ± 4.1	–	–	–
	7α-OH-DHEA	–	–	21 ± 1.6	35 ± 1.8	64 ± 0.9	46 ± 1.1	14 ± 3.3
	7β-OH-DHEA	–	–	7 ± 1.2	14 ± 2.0	36 ± 1.3	52 ± 1.1	46 ± 4.2
KCh J1.3	DHEA	100 ± 0.1	96 ± 0.5	63 ± 3.7	21 ± 3.9	1 ± 1.0	–	–
	7α-OH-DHEA	–	3 ± 0.5	16 ± 2.5	49 ± 1.6	64 ± 0.7	35 ± 3.3	–
	7β-OH-DHEA	–	1 ± 0,2	5 ± 1.2	21 ± 1.8	25 ± 0.5	50 ± 3.2	–
KCh J1.4	DHEA	100 ± 0.2	96 ± 0.5	75 ± 2.1	44 ± 2.7	4 ± 0.6	–	–
	7α-OH-DHEA	–	3 ± 0.1	12 ± 1.1	33 ± 0.9	61 ± 3.2	51 ± 4.3	–
	7β-OH-DHEA	–	1 ± 0.1	4 ± 0.2	13 ± 1.2	24 ± 1.9	36 ± 3.9	–
KCh J1.6	DHEA	100 ± 0.1	87 ± 0.8	36 ± 3.4	4 ± 0.5	1 ± 0.3	–	–
	7α-OH-DHEA	–	8 ± 1.1	41 ± 2.9	66 ± 3.6	54 ± 1.1	26 ± 2.3	–
	7β-OH-DHEA	–	2 ± 0.3	16 ± 2.0	24 ± 1.7	30 ± 2.3	46 ± 4.2	–
KCh J2.2	DHEA	100 ± 0.3	88 ± 2.0	55 ± 3.1	–	–	–	–
	7α-OH-DHEA	–	6 ± 0.8	31 ± 2.7	70 ± 0.9	67 ± 2.0	50 ± 1.6	–
	7β-OH-DHEA	–	2 ± 0.7	11 ± 1.1	26 ± 0.4	29 ± 2.6	47 ± 1.3	–
KCh J2.3	DHEA	100 ± 0.1	97 ± 0.4	90 ± 1.6	79 ± 0.9	–	–	–
	7α-OH-DHEA	–	2 ± 0.4	7 ± 1.3	16 ± 1.7	62 ± 0.3	45 ± 1.5	41 ± 4.3
	7β-OH-DHEA	–	1 ± 0.3	3 ± 0.8	6 ± 2.1	31 ± 0.7	47 ± 2.3	39 ± 2.8
KCh J2.4	DHEA	100 ± 0.6	84 ± 0.9	48 ± 3.2	3 ± 0.6	–	–	–
	7α-OH-DHEA	–	11 ± 2.4	28 ± 4.1	65 ± 1.6	62 ± 1.3	40 ± 1.7	2 ± 1.0
	7β-OH-DHEA	–	5 ± 0.7	10 ± 1.4	21 ± 2.0	32 ± 2.2	42 ± 2.2	13 ± 3.2
KCh J2.6	DHEA	100 ± 0.3	100 ± 0.5	81 ± 4.5	27 ± 3.8	6 ± 2.0	3 ± 1.1	–
	7α-OH-DHEA	–	–	14 ± 3.1	38 ± 4.1	47 ± 1.4	34 ± 1.9	–
	7β-OH-DHEA	–	–	5 ± 2.6	15 ± 3.1	28 ± 1.8	49 ± 2.1	–
KCh KW1.1	DHEA	100 ± 0.2	80 ± 2.1	53 ± 2.8	1 ± 0.6	1 ± 0.8	–	–
	7α-OH-DHEA	–	12 ± 1.3	21 ± 2.6	68 ± 3.9	49 ± 1.3	34 ± 2.9	24 ± 2.7
	7β-OH-DHEA	–	4 ± 0.7	8 ± 1.5	26 ± 0.6	39 ± 0.4	56 ± 3.1	56 ± 3.0
KCh KW1.2	DHEA	100 ± 0.3	95 ± 1.7	68 ± 3.9	–	–	–	–
	7α-OH-DHEA	–	4 ± 0.6	22 ± 2.0	69 ± 0.7	50 ± 1.3	34 ± 2.6	23 ± 2.3
	7β-OH-DHEA	–	1 ± 0.3	8 ± 1.9	27 ± 0.9	40 ± 0.5	56 ± 3.0	57 ± 4.4
KCh RJ1.1	DHEA	100 ± 0.1	100 ± 0.8	97 ± 0.7	27 ± 1.6	26 ± 3.3	–	–
	7α-OH-DHEA	–	–	2 ± 0.2	38 ± 0.9	46 ± 1.7	51 ± 0.2	40 ± 3.0
	7β-OH-DHEA	–	–	1 ± 0.3	16 ± 0.7	23 ± 2.8	46 ± 1.5	57 ± 2.4

Biotransformation conditions: 100 mL of cultivation medium (3% glucose, 1% bacteriological peptone) in 300 mL Erlenmeyer flasks, 24 °C, 150 r/min for 7 day

Data are the average of three independent experiments. Standard errors were indicated

In all the tested strains, DHEA was transformed to the same products but majority of them with similar conversion. However, one of the most effective was chosen for further investigation-*Isaria farinosa* KCh KW 1.1. This strain transformed substrate in less than 24 h and do not degrade products in prolonged time. In the culture of the selected strain, transformations of androstenediol, androstenedione, adrenosterone, 17α-methyltestosterone, 17β-hydroxyandrost-1,4,6-triene-3-one and progesterone were performed. Transformation of another 3β-hydroxy-5-ene steroid, a natural metabolite of DHEA, androstenediol, did not give analogous results as some other filamentous fungi [14, 36]. GC and TLC analysis of samples taken during the screening procedure showed multiple products in very low concentrations. Taking into account the number of products and the suitability of such reactions in industry, conducting the preparative biotransformation was groundless.

**Fig. 4:**
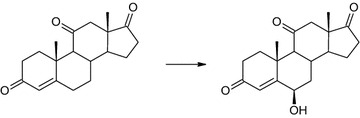
Hydroxylation of adrenosterone by *Isaria farinosa* KCh J2 strain. Biotransformation conditions: 100 mL of cultivation medium (3% glucose, 1% bacteriological peptone) in 300 mL Erlenmeyer flasks, 24 °C, 150 r/min for 7 day

Transformation of androstenedione, a basic steroid with a 4-ene-3-one moiety, resulted in the formation of many unidentified compounds. Analysis of NMR, TLC and GC data ensures that the biotransformation was effective although the quantity of products and their nearly equal and small amounts in the reaction mixture make their identification impossible. It was difficult to extract one main product. Also, another substrate from this group, 17α-methyltestosterone, was transformed into many products. For the same reason as for androstenediol, preparative biotransformation was not carried out. Only adrenosterone was transformed into one main product. The substrate was transformed in about 24 h giving mainly 6β-OH-ADR (Fig. 4). There were still some metabolites that had been isolated, but on the basis of NMR analysis, the steroid structure was not observed. None of them represented more than 10% of the reaction mixture. Compared to the adrenosterone transformation in the culture of *Isaria fumosorosea*, the same product was obtained but much faster (one-day transformation vs. 7 days in *I. fumosorosea* culture) [26]. In contrast to *Aspergillus tamarii*, *Cephalosporium aphidicola*, *Fusarium lini*, *Trichothecium roseum* or *Cunninghamella elegans*, no activity of 17β-hydroxysteroid dehydrogenase or similar enzyme was observed [2, 37, 38].

**Fig. 5:**
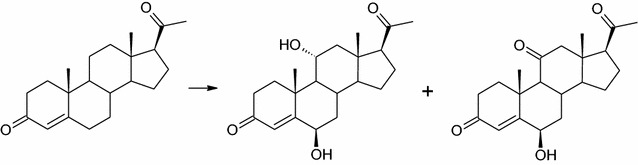
Transformation of progesterone by *Isaria farinosa* KCh KW 1.1 strain. Biotransformation conditions: 100 mL of cultivation medium (3% glucose, 1% bacteriological peptone) in 300 mL Erlenmeyer flasks, 24 °C, 150 r/min for 7 day

Progesterone, the only tested C21 steroid was transformed into two products. 6β,11α-dihydroxyprogesterone (6β,11α-OH-P) was a major product and reached over 70% of the reaction mixture in the first 24 h of the process (Fig. 5). The other one is a result of oxidation of the 11α-hydroxy group, 6β-hydroxy-11-oxo-progesterone (6β-OH,11-oxo-P), and does not exceed 5% of the reaction mixture (Table 3). Products obtained in this reaction are similar to that obtained in transformation of C19 steroid with a 3-one-4-ene moiety, adrenosterone.

**Table 3:** Products’ accumulation during transformation in *Isaria farinosa* KCh KW1.1 culture

Substrate	Compounds found in the reaction mixture (%)	Biotransformation time (days)		
		1	3	7
Adrenosterone	ADR	9 ± 2.6	–	–
	6β-OH-ADR	46 ± 2.8	52 ± 2.2	49 ± 3.6
Progesterone	P	–	–	–
	6β,11α-OH-P	74 ± 1.3	71 ± 2.4	64 ± 3.1
	6β-OH,11-oxo-P	4 ± 0.7	5 ± 1.1	3 ± 0.9
1,4,6-triene-T	1,4,6-triene-T	5 ± 1.3	–	–
	15α-OH-6,7β-epoxy-1-ene-T	37 ± 2.2	35 ± 1.4	32 ± 1.8
	14α-OH-6,7β-epoxy-1-ene-T	20 ± 0.3	19 ± 0.2	16 ± 1.3
	15α-OH-6,7β-epoxy-T	10 ± 0.8	13 ± 2.0	17 ± 2.4

Biotransformation conditions: 100 mL of cultivation medium (3% glucose, 1% bacteriological peptone) in 300 mL Erlenmeyer flasks, 24 °C, 150 r/min for 7 day

Data are the average of three independent experiments. Standard errors were indicated

**Fig. 6:**
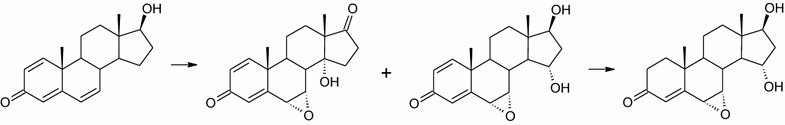
Biotransformation of 17β-hydroxy-androst-1,4,6-triene-3-one by *Isaria farinosa* KCh KW 1.1 strain. Biotransformation conditions: 100 mL of cultivation medium (3% glucose, 1% bacteriological peptone) in 300 mL Erlenmeyer flasks, 24 °C, 150 r/min for 7 day

17β-Hydroxy-androst-1,4,6-triene-3-one (1,4,6-triene-T) is a metabolite of androst-1,4,6-triene-3,17-dione (androstenetrione, ADT), a steroidal aromatase inhibitors used by body-builders. 1,4,6-Triene-T was detected from human and horse urine samples but, to the best of our knowledge, neither detected nor transformed in microbial transformation [39–41]. In this study, the substrate was transformed in the culture of *I. farinosa* KCh KW 1.1 in about 24 h, giving three main products (Fig. 6). All of them are the products of epoxidation of a double bond between C6 and C7. A major amount of product is a result of hydroxylation in the C15 position which gives 15α,17β-dihydroxy-6β,7β-epoxyandrost-1,4-diene-3-one (15α-OH-6,7β-epoxy-1-ene-T) (Table 3). In the resulting compound, the double bond between C1 and C2 is reduced to give 15α,17β-dihydroxy-6β,7β-epoxyandrost-4-ene-3-one (15α-OH-6,7β-epoxy-T). The third isolated product is 14α,17β-dihydroxy-6β,7β-epoxyandrost-1,4-diene-3-one (14α-OH-6,7β-epoxy-1-ene-T), an effect of simultaneous hydroxylation in the 14α position and oxidation of the 17β-hydroxyl group. In the literature, hydroxy derivatives of 17β-hydroxy-androst-1,4,6-triene-3-one were detected by GC/MS or LC/HRMS but their positions were only suggested as a D ring [39–41]. The epoxy derivatives of 17β-hydroxy-androst-1,4,6-triene-3-one were detected for the first time. All obtained compounds were characterised by ^1^H, ^13^C NMR, HMQC, HMBC and are summarised in Table 4.

**Table 4:** ^13^C NMR chemical shifts of products in CDCl_3_

Atom number	Products					
	6β-OH-ADR	15α-OH-6,7β-epoxy-1-ene-T	15α-OH-6,7β-epoxy-T	14α-OH-6,7β-epoxy-1-ene-T	6β,11α-OH-P	6β-OH,11-oxo-P
1	36.10	155.97	36.28	155.69	39.11	36.15
2	34.11	127.25	34.26	127.37	34.55	34.18
3	200.67	185.06	198.47	184.97	201.14	200.56
4	127.17	128.97	129.70	129.36	127.17	127.24
5	166.61	161.23	162.89	160.32	168.41	166.45
6	72.13	56.62	58.31	56.43	73.19	72.52
7	38.10	58.51	55.68	57.24	37.66	38.94
8	30.65	31.89	31.48	38.81	28.50	31.42
9	63.13	51.31	52.06	44.41	59.10	62.51
10	37.68	43.70	36.93	42.02	39.39	37.97
11	207.41	23.10	21.03	21.14	69.02	208.09
12	50.50	37.47	37.54	30.27	50.52	56.76
13	50.44	42.68	43.39	53.38	44.40	47.02
14	49.71	52.12	52.52	80.47	55.44	54.87
15	21.69	68.54	68.43	24.97	24.32	24.09
16	36.03	43.49	43.41	32.93	23.14	23.49
17	217.24	80.84	80.96	216.85	63.27	62.18
18	14.91	13.66	13.59	18.95	14.67	14.47
19	19.32	19.00	16.95	18.67	20.33	19.20
20					209.06	207.99
21					31.45	31.10

## Conclusions

*Isaria farinosa* is a filamentous fungus known for its parasitic activity. These preliminary studies indicate the possibility of using this species as a biocatalyst in the transformation of steroid compounds. The used strains were characterized by their hydroxylation capacity. Dehydroepiandrosterone, adrenosterone and progesterone were transformed to a maximum of two products of each. The 17β-hydroxy-androst-1,4,6-triene-3-one was microbially transformed for the first time, giving three novel metabolites.
